# Driver fatigue detection through multiple entropy fusion analysis in an EEG-based system

**DOI:** 10.1371/journal.pone.0188756

**Published:** 2017-12-08

**Authors:** Jianliang Min, Ping Wang, Jianfeng Hu

**Affiliations:** The Center of Collaboration and Innovation, Jiangxi University of Technology, Nanchang, China; School of Psychology, CHINA

## Abstract

Driver fatigue is an important contributor to road accidents, and fatigue detection has major implications for transportation safety. The aim of this research is to analyze the multiple entropy fusion method and evaluate several channel regions to effectively detect a driver's fatigue state based on electroencephalogram (EEG) records. First, we fused multiple entropies, i.e., spectral entropy, approximate entropy, sample entropy and fuzzy entropy, as features compared with autoregressive (AR) modeling by four classifiers. Second, we captured four significant channel regions according to weight-based electrodes via a simplified channel selection method. Finally, the evaluation model for detecting driver fatigue was established with four classifiers based on the EEG data from four channel regions. Twelve healthy subjects performed continuous simulated driving for 1–2 hours with EEG monitoring on a static simulator. The leave-one-out cross-validation approach obtained an accuracy of 98.3%, a sensitivity of 98.3% and a specificity of 98.2%. The experimental results verified the effectiveness of the proposed method, indicating that the multiple entropy fusion features are significant factors for inferring the fatigue state of a driver.

## Introduction

Detection of driver fatigue using electronic and information technology is a meaningful research topic for driving safety assistance systems [1, 2]. Driver fatigue is one of the most important factors in traffic accidents. After driving for an extensive period, people experience fatigue, which decreases their reaction times during emergencies and contributes to casualty accidents. Some studies reveal that 15%-20% of all fatal traffic accidents are related to driver fatigue, and recent statistics estimate that 1,200 deaths and 76,000 injuries can be attributed to fatigue-related crashes annually [3]. Thus, promoting technologies for the detection or prevention of driver fatigue is crucial.

The emergence of artificial intelligence and the rapid development of electronic and information technology provide opportunities for detecting driver fatigue. Numerous methods, including subjective and objective detection methods, have been proposed in recent years [4–10]. However, recognition rates that are based on subjective detection methods are greatly influenced by a driver’s own judgment or the actions of other drivers, and real-time detection of driver fatigue is difficult. Measurements of driver fatigue based on objective detection methods are categorized as vehicle driving parameters, such as speed, lane tracking, and steering wheel rotation, using electronic sensors [11]; driver behavior characteristics, such as eye closure, blink rates and facial expressions, using video imaging techniques [12, 13]; and driver physiological parameters, such as electrocardiogram (ECG), electrooculogram (EOG), electromyogram (EMG) and electroencephalogram (EEG) [14, 15]. If we aim to detect a driver's fatigue state in advance and do not consider the influence of individual behaviors, light and the angle of image acquisition, the use physiological parameters for detecting driver fatigue is beneficial. For the direct reaction of the brain status, EEG is the most common and effective method for identifying driver fatigue [16].

Various computational approaches based on EEG data have been developed for observing and analyzing driver fatigue [17–20]. Recently, entropy has been extensively applied in the analysis of EEG signals because an EEG signal is a complex, unstable, and nonlinear signal [21–25]. Entropy is a nonlinear measure that reflects the degree of uncertainty in a given system, which enables it to be exploited for measuring the level of chaos of nonlinear time series data, providing distinguishable variation for normal and abnormal brain signals. In recent years, studies based on the measurement of entropies have been expanded in several different fields, and new concepts of entropy, such as sample entropy, approximate entropy, wavelet entropy, log energy entropy, Renyi’s entropy and fuzzy entropy, have been applied. These measures have been employed to analyze physiological time series data, such as fatigue EEG data and other biological data. Xiong characterized driver fatigue level using two types of entropy—approximate entropy and sample entropy—via a support vector machine (SVM) classifier [26]. Chai applied an independent component in an entropy rate-bound minimization analysis to separate EEG fatigue data, AR modeling as features extraction and a Bayesian neural network as a classification algorithm, obtaining an accuracy of 88.2% [27]. Zhang extracted the wavelet entropy and sample entropy of EEG, including the wavelet entropy of EOG and the approximate entropy of EMG, to estimate the driving fatigue stages; the resulting accuracy was high, reaching approximately 96.5%–99.5% using an artificial neural network [28]. However, few studies have employed the multiple entropy fusion analysis method based on EEG signals to study driver fatigue detection, which may be a promising application of EEG-based systems for assessing and analyzing fatigue in driving safety.

**Fig 1:**
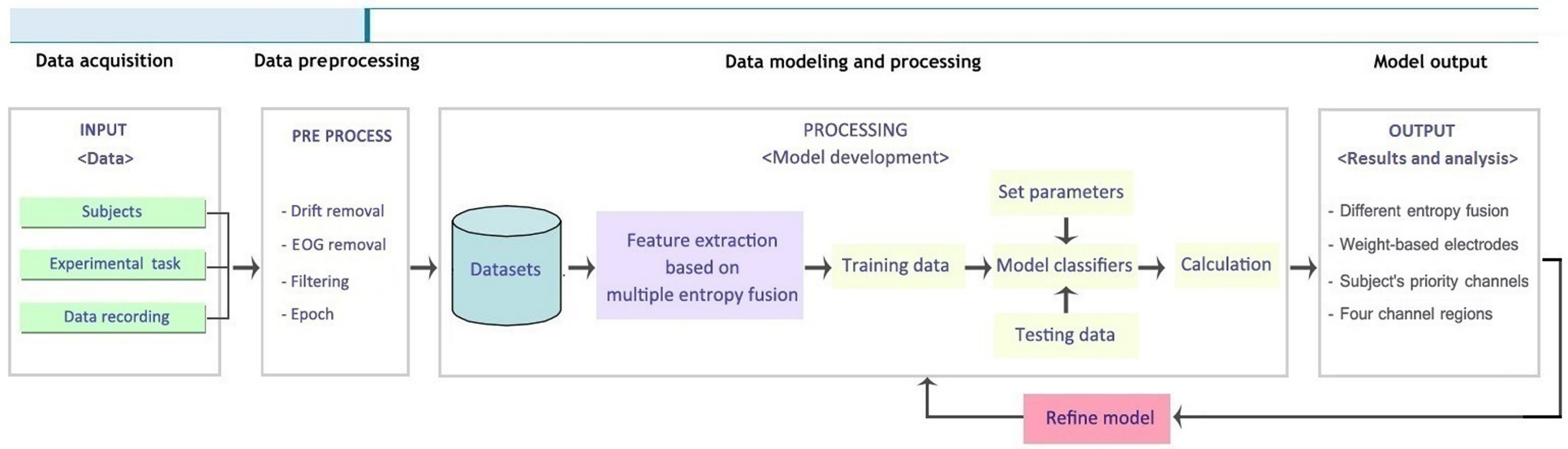
A flowchart to show the operation process of EEG-based fatigue detection system.

In this paper, we present an EEG-based system to effectively detect a driver's fatigue state by analyzing the effects of multiple entropy fusion and calculating four entropies as features. To provide improved performance and a more robust detector of fatigue states, AR modeling as feature extraction was employed in comparison with the multiple entropy fusion method using training and testing data by four common classifiers, i.e., SVM, BP, random forest (RF) and K-nearest neighbor (KNN). According to a simplified channel selection method, we captured four significant channel regions to improve the classification effect using fewer electrodes. An EEG-based system is of potential benefit for driver fatigue detection in relevant areas and may have a complementary role in existing methods. **Fig 1** shows the operation process of an EEG-based fatigue detection system, which primarily includes data acquisition and preprocessing, feature extraction and parameter optimization in data modeling and processing, innovation and results analysis in model outputs.

## Materials and methods

### 1. Subjects and experimental task

Twelve young, healthy men, whose ages ranged from 19–24 years, participated in a highway-driving simulator experiment. All participants were recruited from Jiangxi University of Technology and were asked to refrain from any type of medicine and stimuli, such as alcohol or coffee, during the experiment. The participants were able-bodied persons and had normal sleep time. Prior to the experiment, the participants practiced a driving task for 5 minutes to become acquainted with the experimental procedures and purposes. All experimental procedures were performed with a static driving simulator in a controlled lab environment. This study was approved by the Academic Ethics Committee of Jiangxi University of Technology.

**Fig 2:**
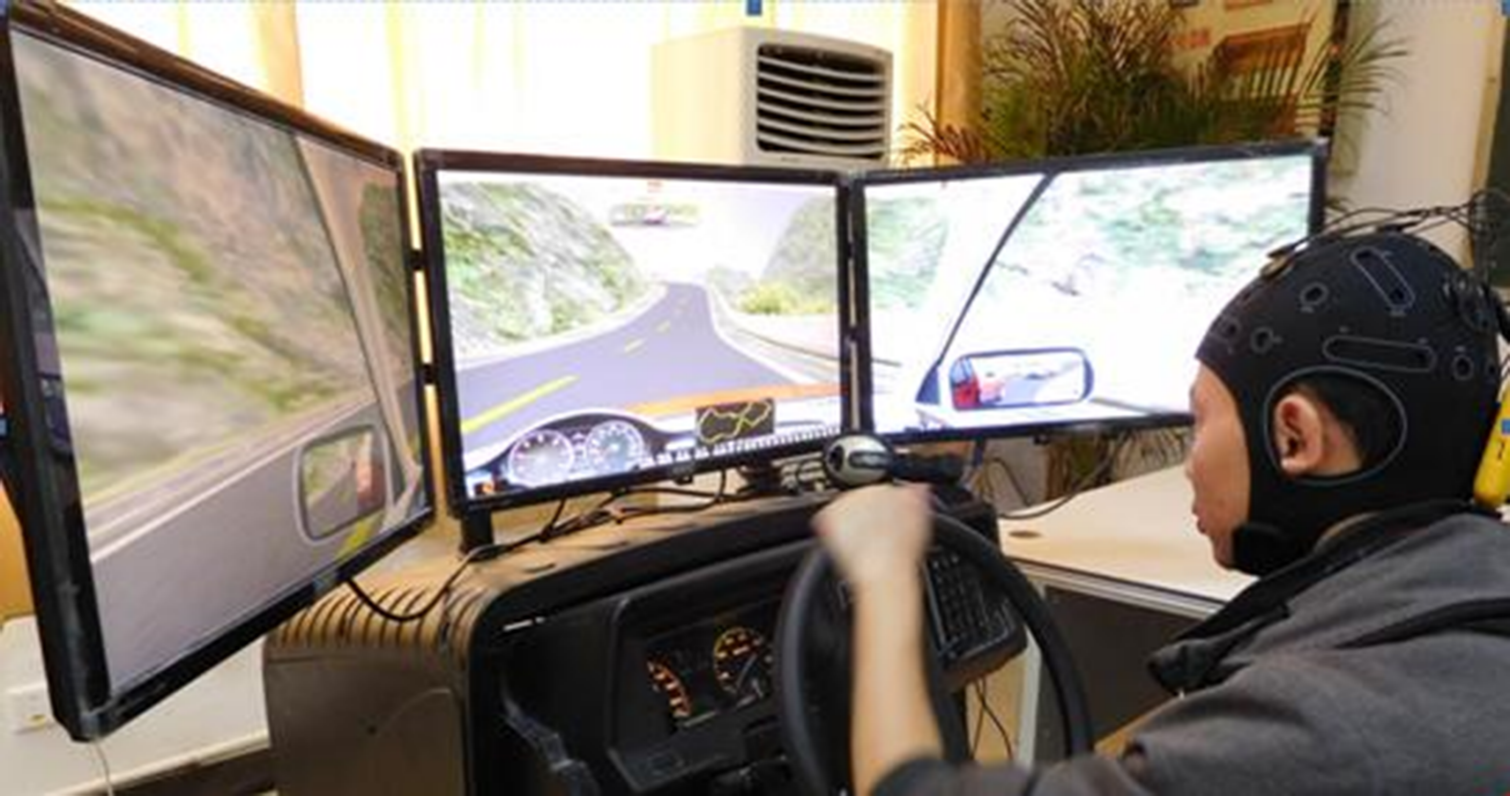
Snapshot of the experimental setup. See the text for further explanation. Notes: the individual in this manuscript has given written informed consent (as outlined in PLOS consent form) to publish these case details.

A sustained-attention driving task was performed by each subject on a static driving simulator (the ZY-31D car driving simulator produced by Peking ZhongYu CO., LTD from Daxing district in Beijing) with a wide screen composed of three 24 inch monitors, as shown in **Fig 2**. On the screen, a customized version of the Peking ZIGUANGJIYE software ZG-601 (car driving simulation teaching system V9.2) was shown. The driving environment selected for this study was a highway with low traffic density; the driving task was started at 9 a.m. After a 5 minute practice session, each subject was given 10 minutes away from the simulator to engage in unconstrained movement within the laboratory. Then, the subjects commenced approximately 1–2 hours of driving after a quick check of all instrumentation.

### 2. Data recording and preprocessing

To record EEG data with two phases, first, when the driving lasted 20 minutes, the last 5 minutes of EEG signals were recorded and labeled as the normal state; second, when the continuous driving lasted 40–100 minutes until the self-reported fatigue questionnaire results indicated that the subject was in a driving fatigue state, the Chalder Fatigue Scale and Li’s Subjective Fatigue Scale [29, 30] were employed, and the last 5 minutes of EEG signals were recorded and labeled as the fatigue state. All channel data were referenced to two electrically linked mastoids at A1 and A2, digitized at 1000 Hz from a 32-channel electrode cap (including 30 effective channels and 2 reference channels) based on an international 10–20 system and stored in a computer for the offline analysis. Eye movements and blinks were monitored by recording the horizontal and vertical EOG signals.

After the EEG signals were collected, the main steps of data preprocessing were performed by the Scan 4.3 software of Neuroscan [31]. The raw signals were filtered by a 50 Hz notch filter and a 0.15 Hz to 45 Hz band pass filter to remove the noise. Then, 5 minutes of EEG data from thirty electrodes were sectioned into 1 s epochs to produce approximately 300 epochs. With the 12 participants, a total of 3600 units of data were formed for the normal state, and 3600 units of data were formed for the fatigue state. The data were randomly and equally divided into training and testing datasets for feature extraction and classification.

Three methods were employed to validate fatigue occurrence: (i) video monitoring of physiological signs of fatigue, such as blinking, head nodding, eye fixation and pupil size, which was verified by EOG analysis of blink rate and eye closure; (ii) performance decrements such as increased crash rates and deviation off the road; and (iii) a self-reported fatigue questionnaire, including the Chalder Fatigue Scale and Li’s Subjective Fatigue Scale, which have been validated in many studies for identifying the fatigue condition [9, 26, 32–35]. The questionnaire included 24 questions, including “Do you have problems with tiredness?”, “Do you need to rest more?”, “Do you have difficulty concentrating?”, “Do you experience blurred vision?”, “Do you feel unresponsive?”, and “Do you feel driving speed changing frequently?” Every question had four choices rated on a four-point scale (-1–2), i.e., better than usual (-1), no more than usual (0), worse than usual (1) and much worse than usual (2). A high fatigue score presented a high level of driving fatigue.

**Fig 3:**
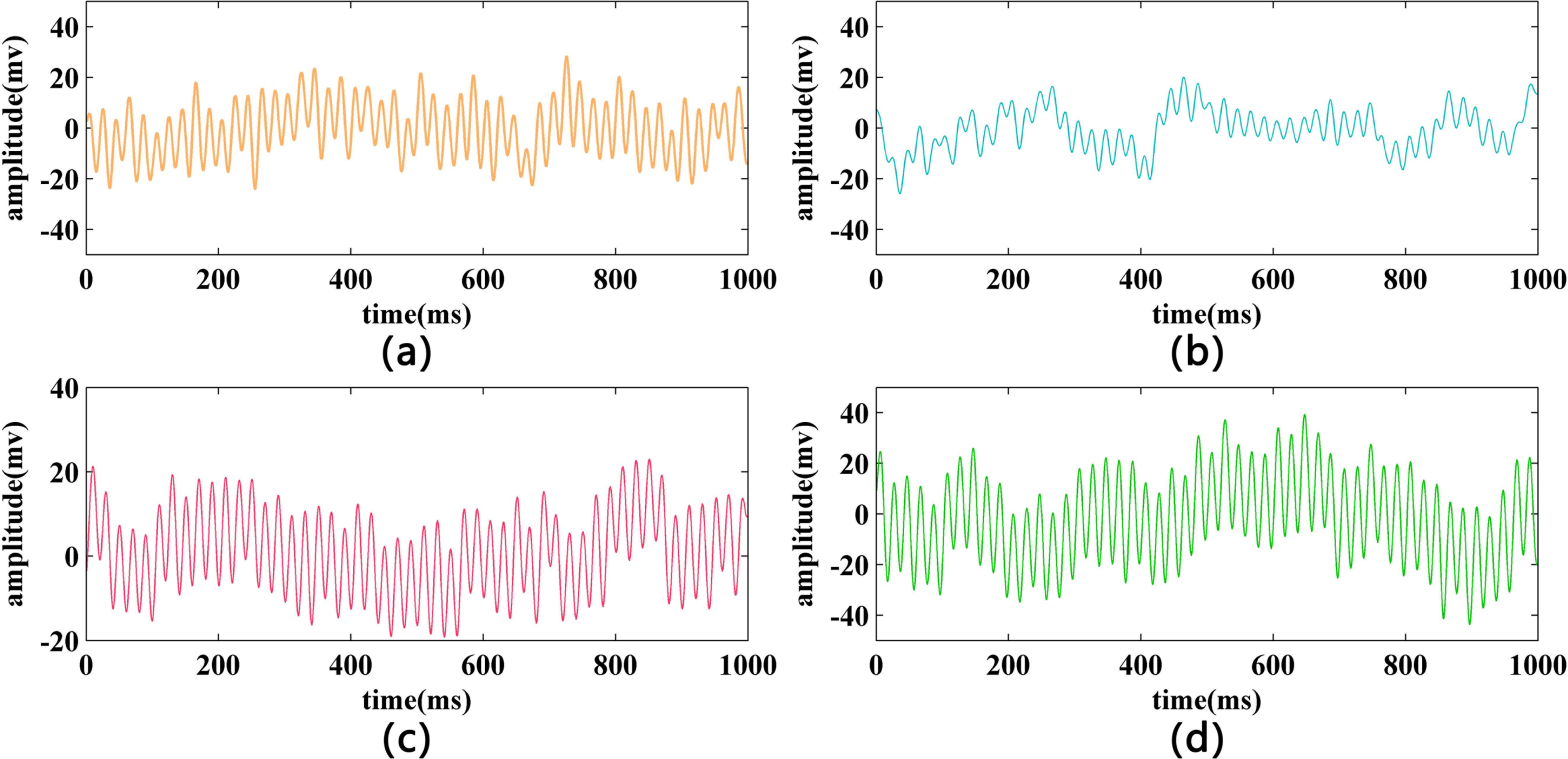
**Sample EEG signals of normal state and fatigue state:** (a) For normal state from FP1 electrode; (b) For normal state from FP2 electrode; (c) For fatigue state from FP1 electrode; (d) For fatigue state from FP2 electrode.

In this process, the EEG signals for two different states (normal and fatigue) were considered. **Fig 3** shows sample EEG signals obtained from FP1 and FP2 electrodes in one trial, which revealed a significant difference between the normal state and fatigue state of the EEG signals. However, the detection of two different states by this visual distinction is difficult. The use of characteristic parameters to detect a fatigue state may be more effective and scientific. Therefore, in this paper, we considered multiple entropies as parameters for detection by calculating four entropies as features based on different electrodes in different trials.

### 3. Feature extraction and classifiers

Entropy is extensively applied to assess the uncertainty of a system. In recent years, various entropies have been expanded in several different fields [36]. As a nonlinear parametric that quantifies the complexity of a time series, entropy can be used to evaluate nonlinear, unstable and dynamic EEG signals [37]. Spectral entropy (PE) is calculated by applying the Shannon function to the normalized power spectrum based on the peaks of a Fourier transform; the calculation algorithm is described in [38]. Approximate entropy (AE) was proposed by Pincus [39]; AE is calculated in a time domain without phase-space reconstruction of a signal and is more suitable for short-length time series data. Sample entropy (SE) was proposed by Richman and Moorman [40]; similar to AE, SE is less sensitive to changes in data length, with larger values corresponding to greater complexity or irregularity in the data. The calculation algorithms of AE and SE are clearly defined in [41]. Fuzzy entropy (FE) can achieve stable results for different parameters and offer better noise resistance using the fuzzy membership function, which is clearly defined in [21]. With an efficient representation of EEG signals, the method using AR model parameters as features by Yule-Walker equations [42, 43] was applied for comparison with the multiple entropy fusion method.

In these four entropies, AE, SE and FE are three ***m*** bedding entropy-based complexity parameters, where ***m*** and ***r*** are the dimensions of phase space and similarity tolerance, respectively. A large similarity tolerance will cause a loss of useful information. However, if the similarity tolerance is underestimated, the sensitivity to noise will be significantly increased. Many studies indicate that the values of **m** and **r** can been selected in two sections: [2, 4] and [0.1, 0.9]. As reported in [44], an **m** of 2 and an **r** of 0.2 times the standard deviation of the time series were the most popular choice when the time series length exceeded 200. In the present study, ***m*** = 2 and ***r*** = 0.2*SD were adopted, where ***SD*** denotes the standard deviation of the time series. Likewise, AR modeling requires the selection of the model order number. The method for selecting the order number in this study will be described later in the paper.

To optimize the detection quality with a time-saving approach, the features were normalized for each participant by scaling between -1 and 1 and adopting min-max normalization. The routine for the normalization process is expressed as follows:

-For all segments,

1. A feature vector is built using the concatenation process, which concatenates the features.
2. The min-max normalization of each feature x_*i*_, *i* = 1,…,n, is computed as follows:
  $$x_{i}^{'}=(newMax-newMin)\frac{x_{i}-x_{\min}}{x_{\max}-x_{\min}}+newMin$$ (1)
  where x_min_ and x_max_ are the minimum value and maximum value in this feature sequence, respectively, and newMin = -1 and newMax = 1.
3. The normalized features are saved.

When the min-max normalization is applied, each feature that lies within the new range of values will remain the same. Min-max normalization has the advantage of preserving all relationships in the data [45]. After concatenating and normalizing the features, a feature-level fusion was explored to improve the detection results. In this study, we applied four classifiers, namely, SVM, BP, RF and KNN. These classifiers are briefly explained.

#### (a) Support vector machine (SVM)

An SVM classifier is a supervised classification technique [46]. The basic idea of the SVM is to transform the data into a high-dimensional feature space and determine the optimal separating hyper-plane using a kernel function. In the case of nonlinear classification, kernels, such as radial basis functions (RBFs), are used to map the data into a high-dimensional feature space. The majority of studies use RBF as the kernel. In this paper, we also used RBF as the SVM kernel function and its two uncertain parameters—the penalty parameter ***c*** and the kernel parameter ***g***—which will be described later in the paper according to a grid search approach [47].

#### (b) Back propagation neural network (BP)

BP is one of the most popular techniques in the field of neural networks [48]. This approach utilizes the methods of mean square error and gradient descent to align the modification to the connection weight of network. Using a neural network toolbox to build a three-layer BP neural network, the input layer contains 120 neurons, the output layer contains one neuron and the number of hidden layer nodes is 20. A Sigmoid function is employed as the transfer function of a hidden layer, and the Levenberg-Marquardt function serves as the training function.

#### (c) Random forest (RF)

RF is a popular machine learning algorithm that has been successfully employed in various fields [49]. RF is a combination of tree predictors, where each tree depends on the values of a random vector that is independently sampled with the same distribution for all trees in the forest. Combining multiple trees produced in randomly selected subspaces has been shown to significantly improve the prediction accuracy. In this study, the number of trees and the number of input variables at each split are 500 and 22, respectively, which is similar to the default values in the MATLAB toolbox.

#### (d) *K*-nearest neighbor (*K*NN)

*K*NN is a supervised learning technique in which a new sample is classified based on *K*-nearest neighbors in the training samples [46]. When determining the classification decision, the class of this new sample depends on the classes of one or several nearest samples, which is related to a very small number of nearest samples. Therefore, a *K*NN classifier may be suitable when many crossing or overlapping samples exist in the datasets.

## Results

### 1. Optimizing parameters and comparisons of entropy at different combinations

**Fig 4:**
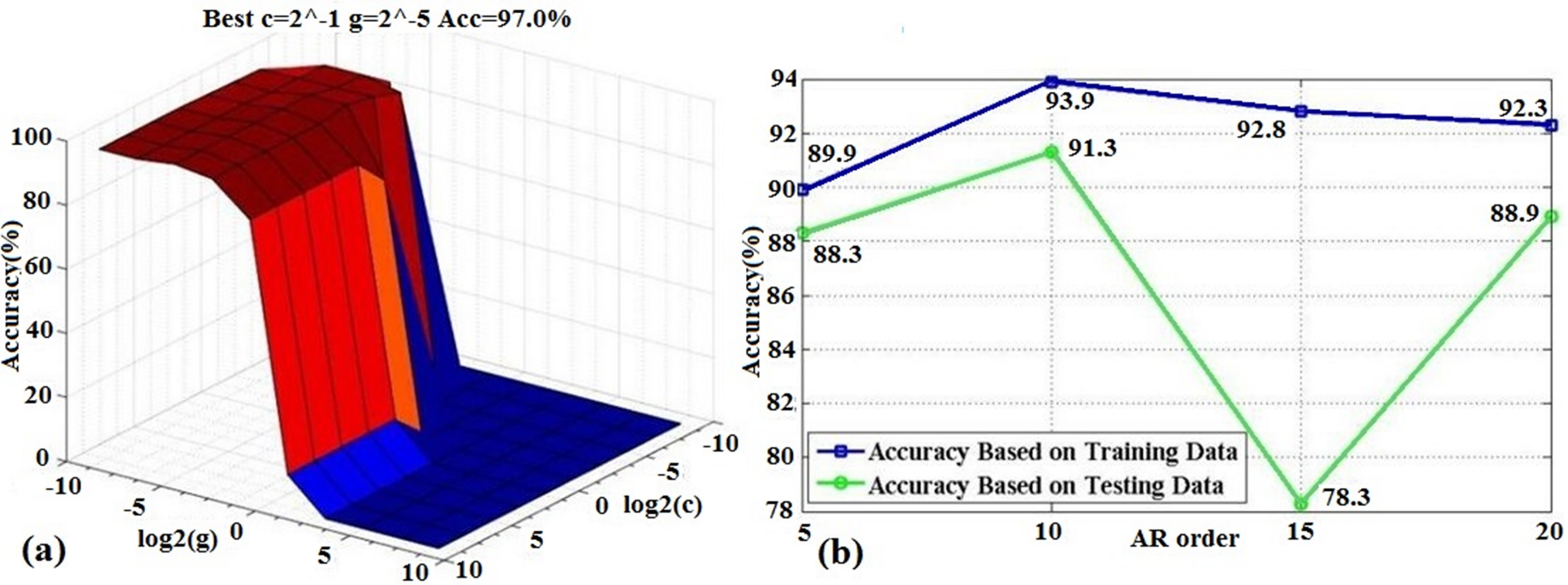
**Using the search method to optimize two parameters of SVM classifier and the order number of AR model with the LOO method:** (a) The optimal values of c = 2^-1 and g = 2^-5; (b) The optimal AR model order number is 10.

For developing a new detector and estimating its potential application value, the detection quality must be properly examined. The leave-one-out (LOO) cross-validation approach is an almost unbiased estimation [50]. The success rate with the LOO method was also used to optimize the two uncertain parameters—***c*** and ***g***—in the RBF-based SVM classifier and select the AR model order number. The obtained results are shown in **Fig 4**, from which (a) when ***c*** = -1 and ***g*** = -5, the SVM classifier reaches its optimized status with the training data, (b) when the AR model order number is 10, the best accuracy is achieved whether using the training data or testing data.

**Fig 5:**
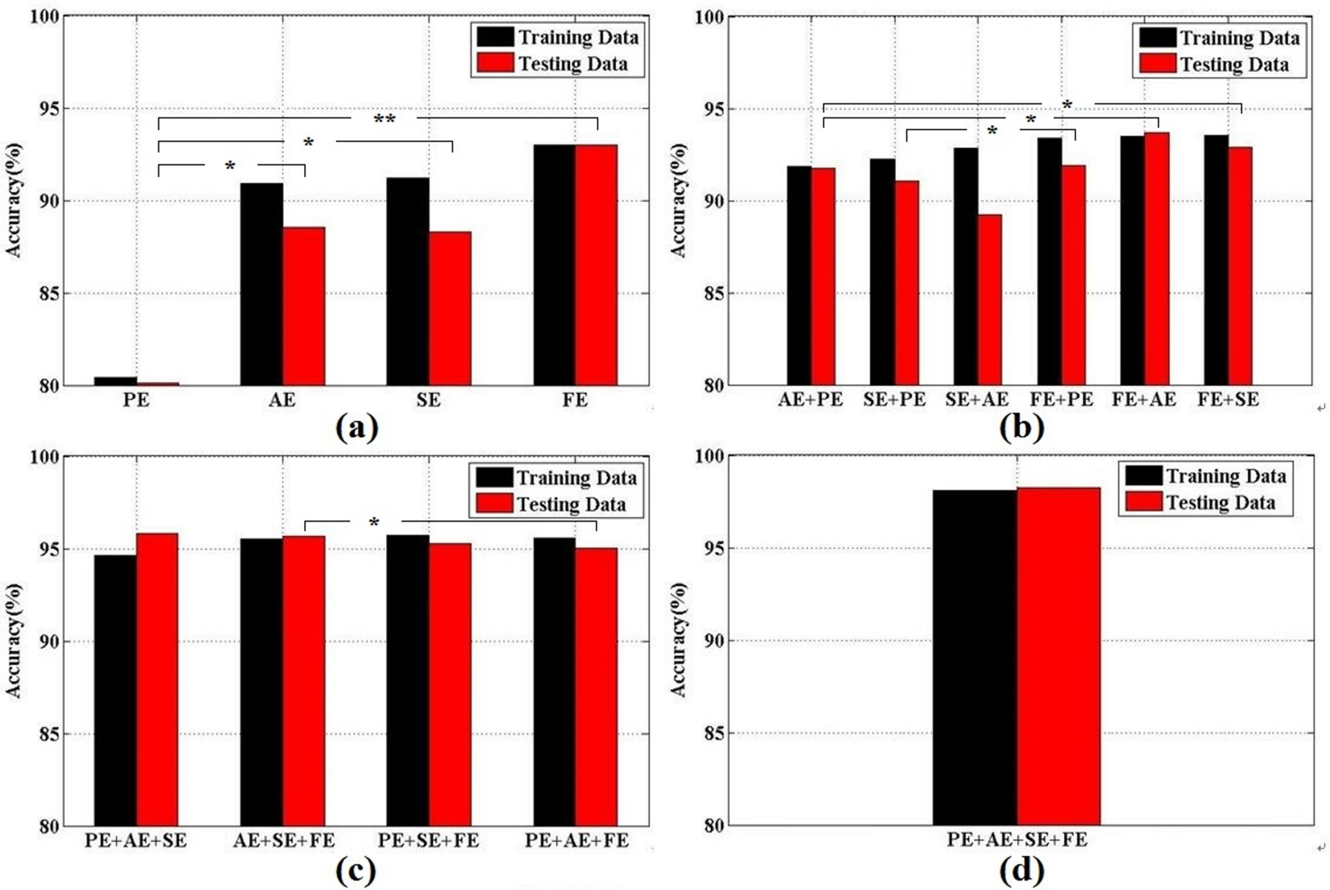
Classification results and performance comparison using different entropy fusion. Statistical analysis refer to the average Acc over all subjects (*p < .05, **p < .01): (a) For using single entropy; (b) For using two types of entropies; (c) For using three types of entropies; (d) For using all entropies.

Compared with the case in which the AR model was used as a feature extractor, the combination of multiple entropies improved the classification performance. To evaluate the performance influence on different combined entropies, we calculated the results of different entropy fusion between PE, AE, SE and FE as features via the SVM classifier. **Fig 5** illustrates the results by showing the average Acc over all subjects for different combined entropies. A significant increase in the mean Acc value was determined based on testing data for a single entropy between PE and AE, PE and SE, and PE and FE in **Fig 5**(A). A significant increase in the mean Acc value was also observed when two or three entropies were fused, as shown in **Fig 5**(B) and **5**(C). **Fig 5** reveals that the multiple entropy fusion method exhibits better performance and robustness.

### 2. Classification and PR-ROC results

In this study, four entropies between normal periods and fatigue periods were calculated for each subject. Results based on training and testing data by four common classifiers were obtained. The well-known performance indicators [51], including the sensitivity (Sn), specificity (Sp) and accuracy (Acc), were employed to evaluate the quantified results. The success rates obtained by the LOO approach for detecting driver fatigue using each classifier based on the training and testing data are listed in **Table 1**. To facilitate the comparison with the multiple entropy fusion method, the corresponding results employing the AR model parameters as features are also given. The highest average Acc of 97.6% was obtained with Sn and Sp: Acc values of 97.6% and 97.6% were obtained based on the training data, and Acc values of 96.8%, 96.4% and 97.0% were obtained based on the testing data. With a BP classifier, Acc values of 96.8%, 97.0%, 96.5%, 92.9%, 93.6% and 92.3% were obtained by employing a BP classifier based on the training and testing data. The corresponding rates were obtained using AR model parameters as features on the same datasets. **Table 1** also demonstrates the better success rates of Acc, Sn and Sp obtained by the SVM, RF and KNN classifier, respectively, which indicates that the performance of detecting fatigue based on the multiple entropy fusion method is not only very high but also very robust.

**Table 1:** Four classifiers performance obtained by fusing entropy feature in comparison with AR parameter feature based on the training and testing data.

Classifiers	Selected features using entropy						Selected features using AR					
	Training Data			Testing Data			Training Data			Testing Data		
	Acc	Sn	Sp	Acc	Sn	Sp	Acc	Sn	Sp	Acc	Sn	Sp
SVM	97.0	96.8	96.7	95.6	95.0	95.7	93.9	93.7	94.1	91.3	92.3	90.2
BP	97.6	97.6	97.6	96.8	96.4	97.0	96.8	97.0	96.5	92.9	93.6	92.3
RF	96.9	96.9	97.0	95.2	95.6	95.0	93.3	93.0	93.5	92.7	92.4	92.9
KNN	95.3	95.1	95.4	94.2	94.3	93.9	85.0	85.9	84.0	84.2	85.6	82.8

**Fig 6:**
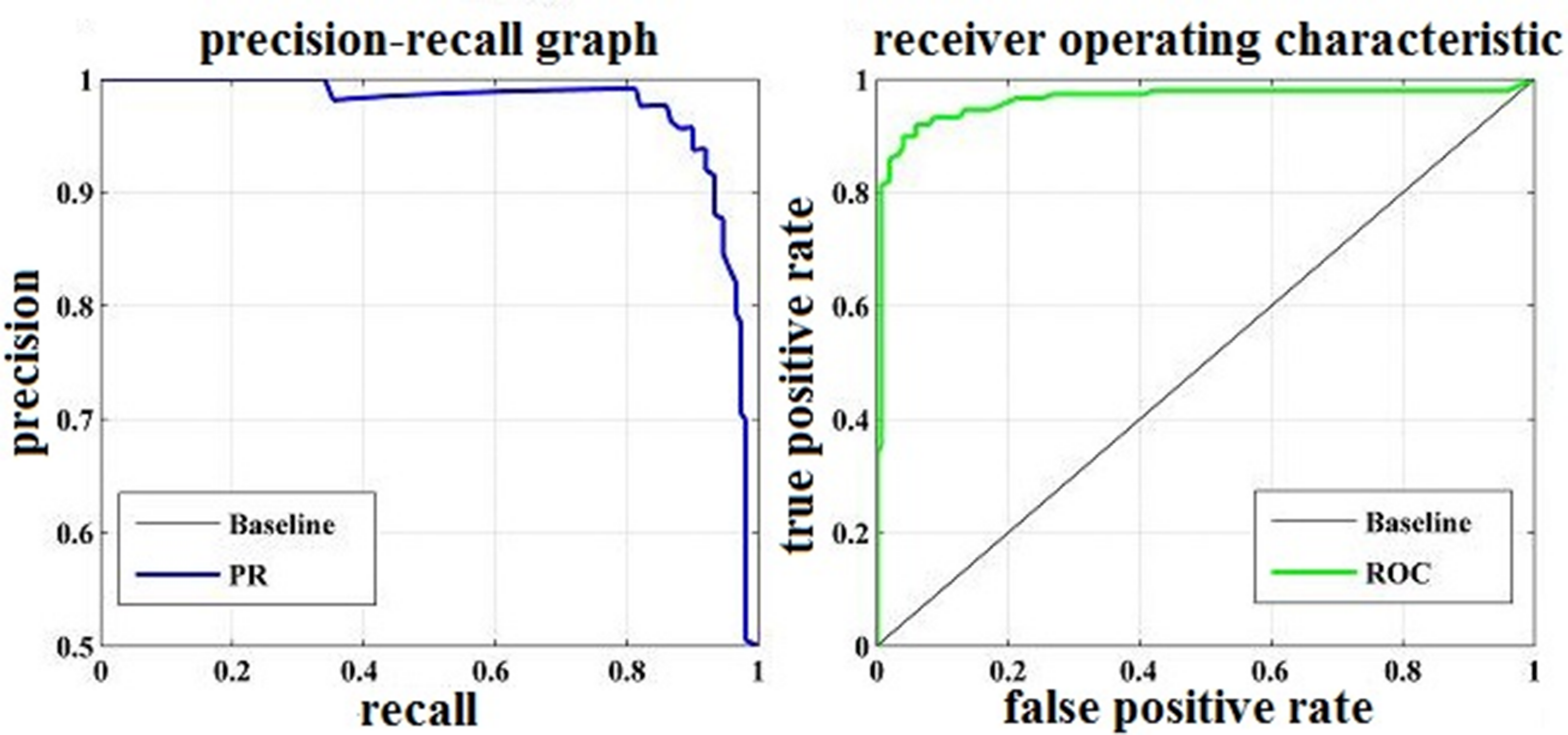
The PR and ROC curves to show the classification quality.

**Fig 6** provides a graphic display of the classification quality for driving fatigue via a precision–recall (PR) curve and the receiver operating characteristic (ROC) curve [52–53]. For the classification in this paper, high precision indicates that a small number of the normal state samples were mislabeled as fatigue state samples, whereas high recall indicates that a small number of fatigue state samples were mislabeled as normal state samples. The ROC curves plot the true positive rate (i.e., Sn) as a function of the false positive rate (i.e., Sp), revealing how the number of correctly classified fatigue state samples varies with the number of incorrectly classified normal state samples. The areas under both the ROC curve and PR curve for detecting driving fatigue in our system represent a distinct improvement, which also indicates that detecting fatigue based on the multiple entropy fusion method is feasible.

### 3. Determination of the significant electrodes

The EEG datasets considered in this study are derived from 30 electrodes of EEG records. Since not all electrodes carry the information of interest, the electrode selection is necessary to decrease computational complexity. In this study, a simplified electrode selection method for calculating the Acc-based weight value of each electrode was proposed to determine the significant electrodes. The main steps for obtaining the weight value V of the *i*-th electrode are illustrated as follows:

1. Calculate the Acc(*i*) of each single *i*-th electrode using the multiple entropy fusion method based on the training data by the SVM classifier.
2. Similar to step 1, after obtaining the accuracy of each single electrode, recalculate the Acc(*ij*), namely, the accuracy of each pairwise combined electrode in all 30 electrodes but only using the *i*-th and *j*-th electrodes to extract the entropy features for obtaining its accuracy, with *i*≠*j* and Acc(*ij*) = Acc(*ji*).
3. Calculate the weight value V of the *i*-th electrode using the following equation:
  $$V_{i}=\frac{Acc(i)+\sum_{j=1,j\neq i}^{30}\left(Acc_{\left(ij\right)}+Acc_{\left(i\right)}-Acc_{\left(j\right)}\right)}{30}$$ (2)
  for *i* = 1 to 30 electrodes.

**Fig 7:**
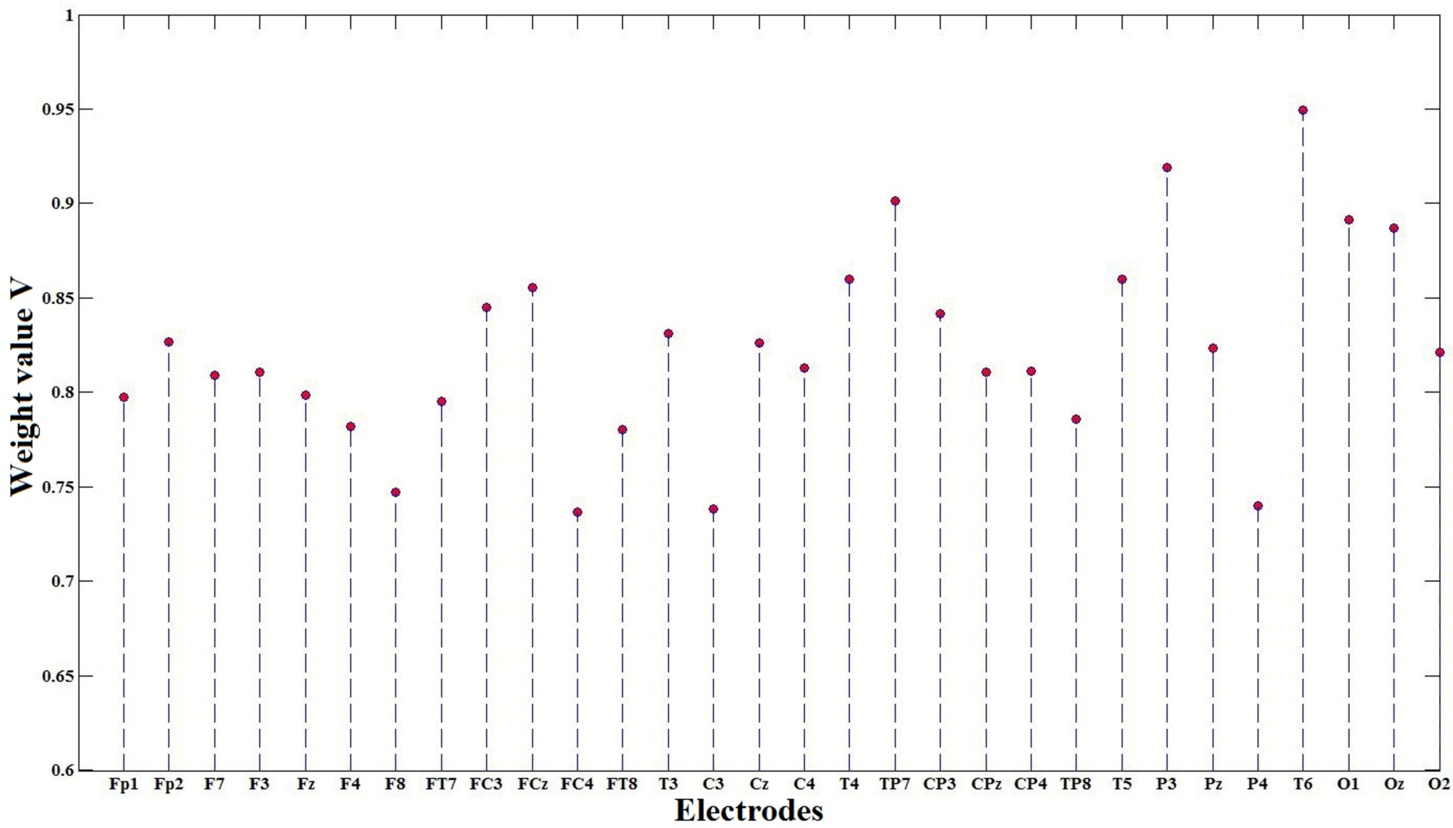
The weight value V of each electrode according to a simplified electrode selection method was plotted.

With this process, the average weight value V of each electrode for each subject was obtained, as shown in **Fig 7**. The variation of different electrodes is distinct. The weights of the T6, P3, TP7 and O1 electrodes is remarkably higher than the weights of the FC4, C3, P4 and F8 electrodes, which indicates that each subject for driving fatigue has different priority channels. Ten types of representative electrodes based on the weight value V for each subject are given in **Table 2**. The T6 electrode attains the largest average weight value of 0.95, and the average weight values of the top ten electrodes exceed 0.84. The top ten electrodes naturally formed four channel regions—A, B, C and D—as clearly discussed for additional analysis below.

**Table 2:** Top ten electrodes based on the weight value V for each subject.

Electrode	T6	P3	TP7	O1	Oz	T4	T5	FCz	FC3	CP3
Sub1	0.91	0.89	0.87	0.95	0.93	0.93	0.89	0.92	0.79	0.89
Sub2	1.01	0.92	1.01	0.98	0.99	0.81	1.01	0.79	0.84	0.90
Sub3	1.06	1.00	1.05	0.81	0.83	0.81	0.80	0.99	0.85	0.75
Sub4	0.99	0.91	1.05	0.88	0.80	0.72	1.05	0.74	0.86	1.02
Sub5	0.89	0.89	0.90	0.95	0.84	0.77	0.98	1.07	1.02	1.06
Sub6	1.03	0.62	0.92	0.67	0.85	1.05	0.85	0.63	0.69	0.59
Sub7	1.09	1.07	0.83	0.77	1.08	1.01	0.97	1.02	0.65	0.52
Sub8	0.68	0.77	0.86	0.82	0.83	0.78	0.90	0.67	1.05	0.73
Sub9	0.68	0.84	0.97	0.58	1.01	0.78	0.73	1.02	1.00	1.00
Sub10	1.13	1.10	0.91	1.12	0.68	0.90	0.54	0.52	1.00	0.80
Sub11	0.97	1.02	0.55	1.11	0.76	0.92	0.60	1.01	0.52	0.77
Sub12	0.95	0.99	0.89	1.07	1.05	0.83	0.99	0.88	0.87	1.06
**Average**	0.95	0.92	0.90	0.89	0.89	0.86	0.86	0.85	0.84	0.84

## Discussion

Entropy is generally accepted as an index for measuring the degree of uncertainty of a given system. As reported in [54], the degree of uncertainty for brain activity signals exhibited significant differences between a normal state and a fatigue state of driving. For the promiscuity and robustness, we presented an EEG-based system to detect driver fatigue by analyzing the effect of multiple entropy fusion according to four common entropies: PE, AE, SE and FE. Considerable progress in distinguishing these states and capturing four channel regions has been achieved with the use of detectors employed in the study of brain entropy.

To obtain the characteristics of a time series for classification, four common entropy measures are computed from EEG brain signals. PE uses the power spectrum of a signal to estimate the regularity of a time series. Oscillation rhythms with a pattern of PE can emerge due to a change of signals and support real-time monitoring by the low-load computation of PE. Compared with PE, AE requires a shorter data segment input for calculation and has certain noise immunity. AE measures the randomness or regularity of a time series in multiple dimensions and is extensively applied in the field of EEG analysis. The algorithm of SE is similar to the algorithm of AE. SE measures the predictability of consequent amplitude values of the EEG based on information about previous amplitude values, and its calculation is not dependent on the length of the data. The low sensitivity of SE to missing signal data produces a very small effect on the calculated value of SE. Rigas [55] proposed a methodology that fused a set of features, including PE and AE, for recognition of a driver’s state and yielded a maximum detection accuracy of 88%. Even when Chen [56] adopted the fusion of four nonlinear methods by extracting PE, AE, SE and Renyi entropy from EEG and a blink feature from EOG, the accuracy was only 97.3%. In our paper, we employed PE, AE, SE and FE as features to detect driver fatigue. The results are satisfactory: an accuracy of 98.3%, a sensitivity of 98.3% and a specificity of 98.2%. **Fig 5** reveals that FE is the most important measure in detecting driver fatigue in our EEG-based system, with some significant differences among measures. Using a fuzzy membership function for computing the vector similarity, FE offers better noise resistance and is extensively employed in many fields [57]. Compared with existing research methods for driver fatigue [58], the entropy measure is a useful tool for EEG analysis because it can consider the effectiveness and robustness of an offline analysis in a real-time driving environment in the future.

Future systems should utilize wearable devices with fewer electrodes to provide fatigue warnings, which will affect the comfortableness and convenience of a system. Thus, electrode selection optimization should be applied to system algorithms to improve their quality of detection. For optimal channels, the Acc-based weight value calculated by **Eq 2** should be employed. The top ten significant electrodes for each subject are listed in Table 2, namely, T6, P3, TP7, O1, Oz, T4, T5, FCz, FC3 and CP3. Xiong [26] classified driving fatigue and noted significant differences both for AE and SE on P4, P3, Pz and Oz. As reported in [59], the highest correlation was observed for channels Oz and O1, which were employed to improve the system performance of drowsiness detection. The ability to optimize electrode selection in advance of analyzing the electrodes involved in the system computation provides an opportunity to decrease computational complexity. Several researchers have adopted an optimal electrode strategy for determining the significant electrodes. Li [17] introduced a grey relational analysis to determine the relative optimal indicator from the alternative indicators of driver fatigue, which treated Fp1 and O1 as the significant electrodes. Lee [60] also captured eight-channel EEG readings based on the highest mutual information value for predicting the fatigue level via Bluetooth wireless communication. Compared with their study, we reduced the noise sensitivity, which rendered the multiple entropy fusion method stronger and robust in detecting fatigue. From the standpoint of computational efficiency, the computational expense of our study is less than that of the method described by Lee [60].

In addition to the variation of the different electrodes listed in **Table 2** and **Fig 7**, we are interested in the brain regions in which these select channels are located. Due to the importance of EEG-based multi-channel analysis, complicated behaviors from time series are observed and some information is extracted in complex systems. Similarly, numerous multi-channel time series analyses and EEG analyses have been performed; refer to [61, 62]. Using a wavelet transform and k-means clustering, Gurudath [63] captured EEG signals for drowsy driving detection via a multi-channel electrode system. Combining adaptive optimal kernel time-frequency representation and a visibility graph, Gao [64] characterized the topological structure of the networks generated by EEG signals for detecting epileptic seizures. In our paper, the selected electrodes in each subject were mapped onto their corresponding locations in the electrode cap, which also used channel topography for multi-channel analysis.

**Fig 8:**
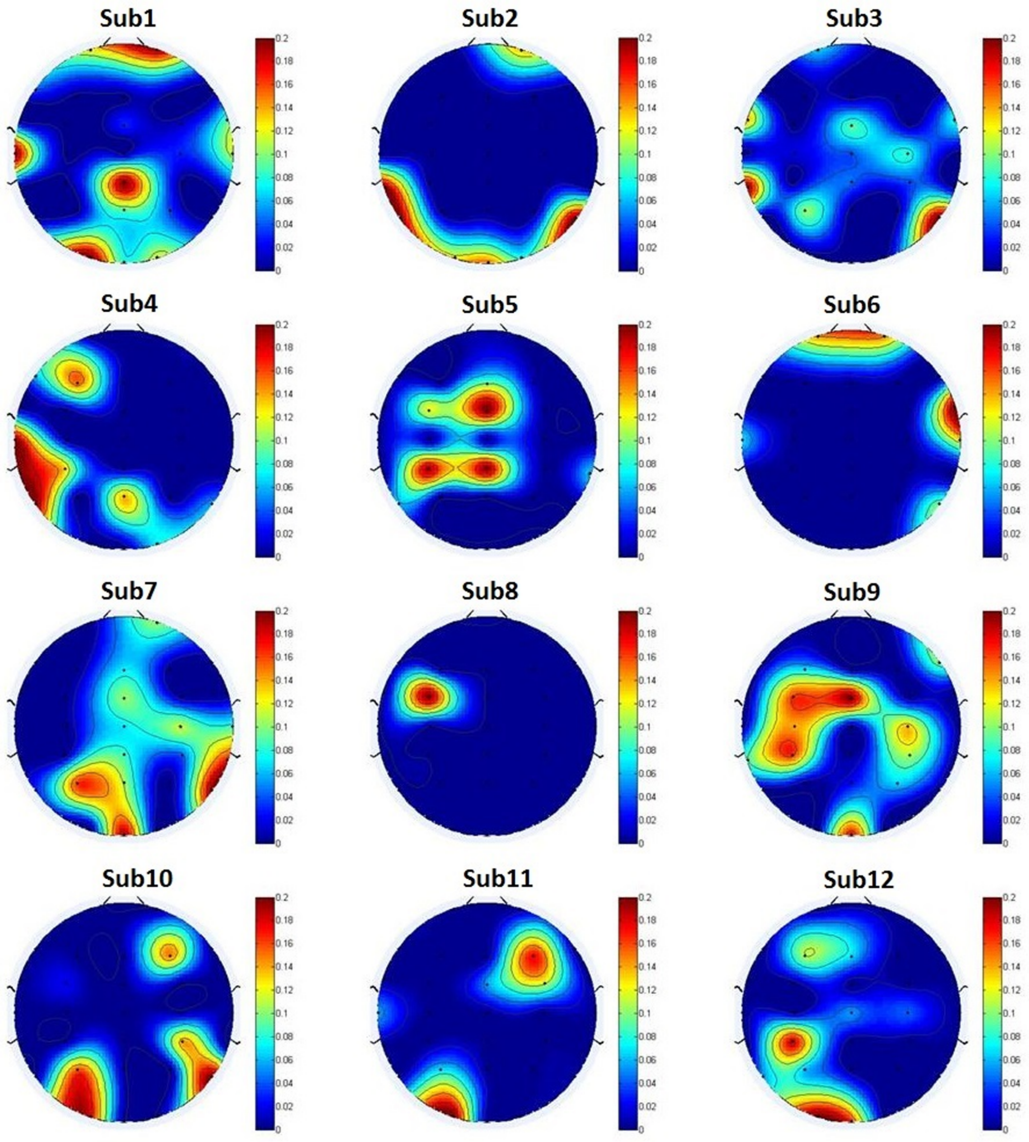
The weight-based channel topographies of twelve subjects. Topographies demonstrate that the subject’s priority channels according to the weight value V locate over which part of the cortex area. The color indicates the importance of a channel in our classification, and the importance of a channel is determined by its weight value V for each subject. The **Sub** is the abbreviation of subject.

**Fig 9:**
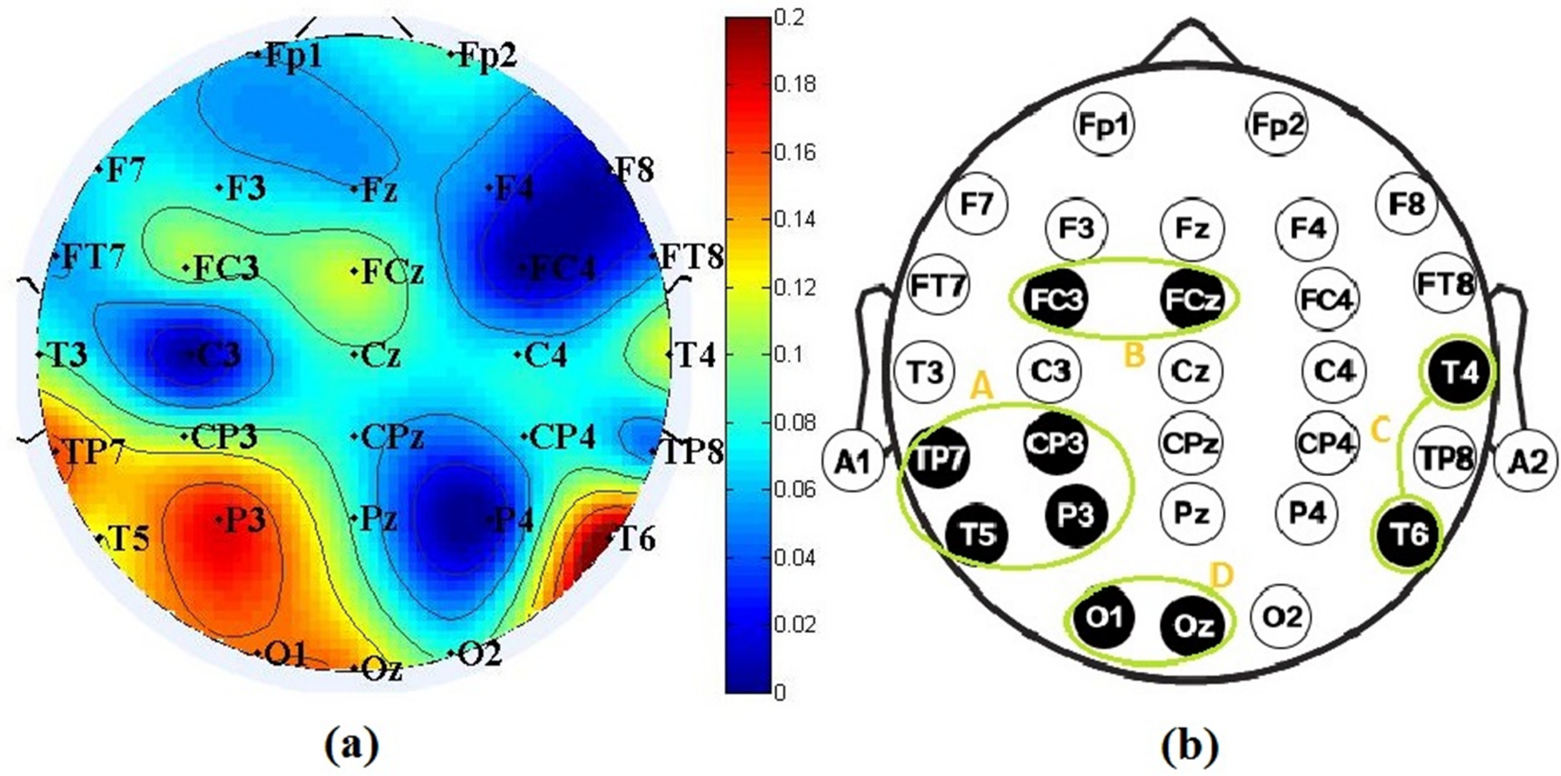
**A graphic comparison of four channel regions A, B, C and D formed by the top ten weight-based electrodes in Table 2**: (a) A topographic mapping of describing channel regions; (b) A black and white diagram of simplifying channel regions.

**Fig 8** presents the weight-based channel topographies of twelve subjects. The colors indicate the weight values of 30 electrodes as determined by **Eq 2**. Note that the values were subjected to the standard conversion in **Eq 1** for scaling between 0 and 1. Then, the uniform value was set to zero when the value V<0.8; otherwise, 0.8 should be subtracted from the uniform value because the average weight values of the top ten electrodes exceed 0.84, as listed in **Table 2** (but retaining one decimal fraction in this case). As shown in **Fig 8**, for the larger weight value, the selected electrodes are primarily located over certain parts of the cortex area for each subject. For example, the left and middle posterior regions have significant differences displayed in Sub1, 2, 7, 10, 11 and 12, and the deep color of Sub1, 3, 6, 7 and 10 also emerged in the right central region. The distribution of selected significant electrodes is not significantly scattered. An interpretation of the distribution in the EEG brain regions is provided in Borghini’s work [65], which reveals that these signal bursts were more dominant in the central and posterior EEG channels during the monotonous driving task. From the standpoint of EEG rhythms, delta and theta rhythms built up as fatigue increased, especially in the frontal and central areas, and beta rhythms increased as alertness decreased, especially in the posterior regions [66]. To evaluate the influence of the selected electrodes according to the weight-based channel selection method, the average weight values of 30 electrodes for 12 subjects are also used to draw the brain topography shown in **Fig 9**(A). The deep colors that are primarily distributed in these four regions, that is, the A, B, C and D regions, are simplified in the black and white diagram shown in **Fig 9**(B). Similar gains of regional brain wave activity regarding fatigue can be found in the work of Craig [67] and Simon [68]. In the diagram, the A and D regions describe the left and middle posterior regions, respectively; the C region describes the right central region; and the B region is the middle central region that emerged in Sub3, 5, 8, 9 and 12 in **Fig 8**. The selected significant electrodes in these regions are the top ten electrodes listed in **Table 2**. In addition, the Fp2 electrode may have potential applications when we use only one electrode to detect fatigue level in real driving situations due to its deep color in Sub1, 2, 6 and 7 in **Fig 8** and **Fig 9**(A). This finding demonstrates the effectiveness of the weight-based channel selection method. Although **Figs 8 and 9** revealed significant results, a causation between the EEG activity of brain domains and driver fatigue was not observed due to the complexity of the human brain and individual differences. The correlation of EEG activity or brain lobes with driver fatigue should be investigated in future research with a very rigorous study protocol and authoritative clinical medical evidence. Our findings suggest that rigorous and meticulous work should be continued in subsequent studies.

**Table 3** lists the classification results of the selected channel regions A, B, C and D obtained by our proposed method and the randomly selected region R; a comparison with these regions was performed based on the weight channel selection method. The region R selected two adjacent electrodes each time; its results are the average results of the randomly selected region for ten times. The highest average Acc of 98.3% was achieved with Sn and Sp of 98.3% and 98.2%, respectively. The Acc achieved by a BP classifier based on the training data using four electrodes in region A was higher than the Acc obtained using all electrodes in **Table 1**. The success rates of other regions by our four classifiers yielded the same or better classification results using fewer electrodes. We can conclude that the results of region R obtained poor performance with a decrease of a few percent, which implies that our method can achieve satisfactory performance using multiple entropy fusion with few electrodes based on the subject’s priority channels.

**Table 3:** Classification results of the selected channel region based on the weight value V using proposed methods.

Classifiers	Region A			Region B			Region C			Region D			Region R		
	Acc	Sn	Sp	Acc	Sn	Sp	Acc	Sn	Sp	Acc	Sn	Sp	Acc	Sn	Sp
SVM	96.7	96.2	96.9	94.0	92.8	95.2	92.2	91.7	93.4	93.5	93.9	93.1	87.2	86.0	87.7
BP	98.3	98.3	98.2	96.7	97.0	96.3	96.8	96.7	96.9	95.3	95.5	95.1	89.3	90.0	89.6
RF	96.4	96.9	96.0	93.7	92.9	94.5	94.0	93.6	94.4	93.3	94.2	92.5	86.7	86.9	86.3
KNN	93.7	93.0	94.2	91.4	90.2	92.7	90.5	90.7	89.9	90.7	90.9	91.3	85.7	85.5	86.3

In recent years, several research groups have addressed this problem using EEG signals to study driver fatigue detection. The related classification performance adopted in their studies, which is listed in **Table 4**, implies that our results based on fewer features of four electrodes were better than the results of other several classification methods. Detecting driver fatigue in traffic safety has a direct application for warning of driving fatigue, reducing excessive driving and reducing casualties. Future research is recommend based on these two aspects: (i) the variability of fatigue data over time and (ii) the real-world application of an EEG-based fatigue monitoring system. The time variability of fatigue data should help to measure the uncertainty in the data variables of a given system and capture the sensitivity of the obtained results [73, 74]. We performed an offline analysis on EEG datasets recorded from online experiments in this study. Because the offline and online classifications have distinct characteristics, an additional study in a real-time online experimental environment should be conducted to confirm our findings. Real-time fatigue-level detection with a wireless EEG device, such as a smartphone, tablet or cloud server, can be extensively employed in the future. Thus, a platform for a mobile fatigue monitoring system that satisfies the requirements of real-time online modality is needed. A global sensitivity and uncertainty analysis of a driver fatigue monitoring model/system would be useful to capture the variability of the detection results in the future [75, 76]. According to **Figs 8** and **9**, different subjects have individual differences, which causes different subjects to have different priority regions. To consider the individual differences and the convenience of actual application of the method, we can use different priority channel regions to select few electrodes to detect and alert driving fatigue in real-time driving conditions, which would benefit a driving safety assistance system.

**Table 4:** Performance comparison of the previous works.

RESEARCH GROUPS	METHOD	ACC (%)
Correa [1]	Multimodal Analysis	83.6
Xiong [26]	Approximate Entropy and Sample Entropy	90.0
Chai [27]	Entropy Rate Bound Minimization Analysis	88.2
Zhang [28]	Entropy and Complexity Measure	96.5
Yin [33]	Fuzzy Entropy	95.0
Ko [69]	Fast Fourier Transformation	90.0
Wang [70]	Power Spectral Density	83.0
Mu [71]	EEG Frequency Ratio	85.0
Nugraha [72]	Emotiv EPOC+	96.0
This paper	Multiple Entropy Fusion	98.3

## Conclusions

In this paper, an objective approach based on multiple entropy fusion analysis was proposed to detect driver fatigue in an EEG-based system. To prove the effectiveness and robustness of the proposed method, four common classifiers were actuated for training and testing data, multiple entropy fusion for feature extraction was adopted, and a simplified channel selection method was used for optimizing electrodes. The results indicated that this type of system has potential for detecting driver fatigue since it achieved high success rates with only four electrodes. The feasibility of an EEG-based system for driver fatigue detection in relevant areas or at least a complementary role with existing methods is anticipated. However, some issues need to be resolved in the future. The experiment must be validated with a larger pool of participants and real-world driving EEG data. Future research should focus on analyzing the efficiency and convenience of different frequency bands using entropies for the real-time detection of driver fatigue.
